# The germline landscape of pituitary adenomas: established and emerging predisposition genes

**DOI:** 10.1210/clinem/dgag195

**Published:** 2026-05-16

**Authors:** Edward Mignone, Alexandra Sorvina, David J Torpy, Hamish S Scott, Sunita M C De Sousa

**Affiliations:** School of Medicine, Adelaide University, Adelaide, South Australia 5000, Australia; Department of Endocrinology, Flinders Medical Centre, Bedford Park, South Australia 5042, Australia; Centre for Cancer Biology, SA Pathology and University of South Australia, Adelaide, South Australia 5000, Australia; School of Medicine, Adelaide University, Adelaide, South Australia 5000, Australia; Endocrine & Metabolic Unit, Royal Adelaide Hospital, Adelaide, South Australia 5000, Australia; School of Medicine, Adelaide University, Adelaide, South Australia 5000, Australia; Centre for Cancer Biology, SA Pathology and University of South Australia, Adelaide, South Australia 5000, Australia; Department of Genetics and Molecular Pathology, SA Pathology, Adelaide, South Australia 5000, Australia; School of Medicine, Adelaide University, Adelaide, South Australia 5000, Australia; Endocrine & Metabolic Unit, Royal Adelaide Hospital, Adelaide, South Australia 5000, Australia; South Australian Adult Genetics Unit, Royal Adelaide Hospital, Adelaide, South Australia 5000, Australia

**Keywords:** pituitary adenoma, pituitary neuroendocrine tumor, genetic, germline

## Abstract

Pituitary adenomas are increasingly recognized to have a germline genetic component in a subset of patients, particularly those with young-onset disease, familial clustering or syndromic features. The spectrum of germline variants implicated in pituitary tumorigenesis has broadened considerably, with evidence of both established predisposition genes and a growing number of emerging candidate genes. Established germline predisposition genes—namely, *MEN1*, *PRKAR1A*, *AIP*, *CDKN1B, GPR101*, *SDHx*, and *MAX*—remain central to our understanding of familial pituitary adenoma predisposition and have defined roles in specific clinical contexts which influence adenoma phenotype, age at presentation, surveillance strategies, and family screening. Beyond this, a set of less prevalent variants in other genes—for example, *CABLES1, CDH23, PAM*, *CHEK2*, and the mismatch repair genes—are emerging as potential contributors, although the pathogenicity and clinical relevance of these genes remain to be fully established. Identifying causative germline variants in people with pituitary adenomas offers the opportunity of personalized care via gene-specific surveillance strategies, prognostication, cascade testing, and reproductive planning to the potential benefit of the individual as well as their families. In this review, we provide a clinically orientated overview of the established and emerging genes implicated in the germline predisposition to pituitary adenomas. We also present a contemporary clinical approach to germline genetic testing in patients with pituitary adenomas.

Located within the sella turcica, the pituitary gland is a complex, hormonally active organ from which pituitary adenomas arise, representing one of the most common intracranial neoplasms (1). The fifth edition of the World Health Organization classification of endocrine and neuroendocrine tumors (NETs) of the pituitary gland published in 2022 termed these lesions pituitary NETs (PitNETs; also referred to as pituitary adenomas [PAs]), subclassified by cell lineage (PIT1, TPIT, and SF1), hormone expression (PRL, GH, TSH, ACTH, LH, and FSH), and associated characteristics (eg, granulation pattern) (2).

While only 5% of all PAs are considered to have a germline genetic basis, these heritable adenomas remain prominent because of their striking young-onset and/or syndromic phenotypes, oftentimes aggressive tumor behavior and treatment refractoriness, and familial presentations. Established PA germline predisposition genes comprise *MEN1*, *PRKAR1A*, *AIP*, *CDKN1B, GPR101*, *SDHx*, and *MAX*, with gene-specific implications for diagnosis, surveillance, family screening, and clinical management. A broader group of emerging candidate genes has also been described, including *CABLES1*, *PAM*, *CHEK2*, mismatch repair (MMR) genes, *PRLR*, *FGFR1*, *AHR*, *USP8*, *PRKACB*, and *KDM1A,* although the level of supporting evidence and the extent of their clinical relevance vary across these associations. Germline contributors to PA predisposition are distinct from the somatic alterations found in sporadic PAs, which are more frequent but with unclear utility in routine clinical practice.

This review aims to provide a contemporary overview of established and emerging PA germline predisposition genes as well as a suggested clinical approach to germline genetic testing in suspected familial PA conditions. The somatic genetic basis of PAs is beyond the scope of the present review but are summarized elsewhere (3, 4).

## Established germline predisposition genes

**Figure 1:**
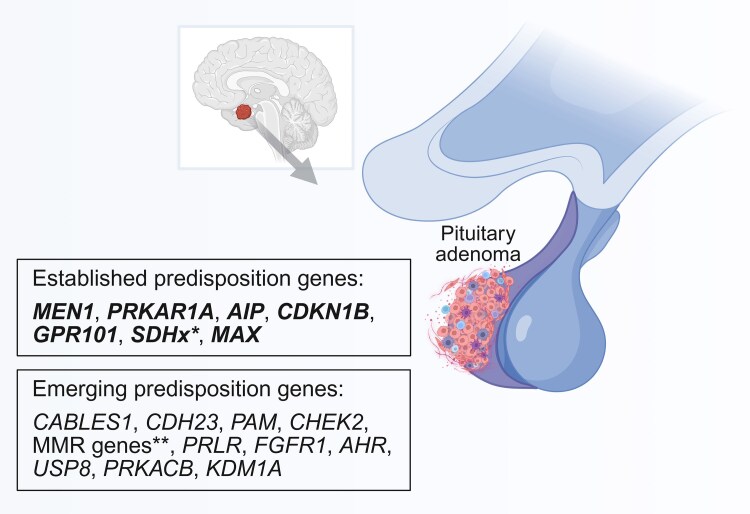
Schematic diagram of established (bold) and emerging (unbold) pituitary adenoma predisposition genes. *SDHx is a collective term for *SDHA*, *SDHB*, *SDHC,* and *SDHD* encoding the subunits of succinate dehydrogenase (SDH), and *SDHAF2* encoding SDH assembly factor 2 protein. **MMR genes refer to the genes involved in mismatch repair, ie, *MLH1*, *MSH2*, *MSH6*, *PMS2* and *EPCAM*.

Established germline associations with PA formation comprise *MEN1*, *PRKAR1A*, *AIP*, *CDKN1B, GPR101*, the *SDHx* genes, and, most recently, *MAX* (Fig. 1). Familial PA conditions may be divided into multiorgan tumor predispositions versus familial isolated PA (FIPA). FIPA is an autosomal dominant disorder defined by the presence of PA in 2 or more family members without clinical or genetic features of other PA predisposition syndromes (5). *GPR101* and *AIP* are associated with isolated PAs, whereas *MEN1*, *PRKAR1A*, *CDKN1B*, *SDHx*, and *MAX* are associated with PAs in addition to gene-specific extrapituitary neoplasms. Apart from the special scenario of *GPR101*-containing duplications which cause X-linked acrogigantism (X-LAG) through ectopic enhancer-induced pituitary misexpression of *GPR101*, each of the PA germline predisposition genes has apparent tumor suppressor functions and is associated with an autosomal dominant predisposition to PA development via germline heterozygous loss-of-function (LOF) variants that may be familial or arise *de novo*. In addition, postzygotic variants resulting in mosaicism have been described in *MEN1* (6), *PRKAR1A* (7), *CDKN1B* (6), *GPR101* (8), and *SDHB* (9). The individual features of the established PA germline predisposition genes follow subsequently in chronological order of their discovery.

### MEN1

Inactivating variants of the *MEN1* gene (Chr 11q13.1, encoding menin) were the first established cause of familial PAs, identified in 1997 through positional cloning (10). Menin is a scaffold protein with tumor suppressor functions, including control of cell proliferation, cell migration, gene expression, and DNA damage repair (11). Germline *MEN1* variants produce the MEN1 syndrome, predisposing to parathyroid adenomas and hyperplasia, PAs, and duodenopancreatic NETs as the cardinal tumors, in addition to a range of other manifestations including angiofibromas, collagenomas, lipomas, adrenal tumors, and gastric, thymic, and bronchopulmonary NETs (12).

Notably, PA is the presenting feature of MEN1 syndrome in 15% to 30% of individuals, with a mean age of onset of approximately 35 years (13). More broadly, MEN1 syndrome rarely manifests before age 5, although 14% of affected children exhibit disease manifestations by age 10 (14). Increased PA risk within MEN1 has recently been associated with prior primary hyperparathyroidism, female sex, and non-missense *MEN1* pathogenic variants (15), although MEN1 is generally considered to lack genotype–phenotype correlations (16). The majority of MEN1-related PAs are prolactinomas, followed by nonfunctioning PAs, and to a lesser extent somatotrophinomas and corticotrophinomas (3, 17). Contrary to the historical association between germline *MEN1* variants and PA treatment resistance, recent data indicate that dopamine agonist response rates are comparable between *MEN1*-associated prolactinomas and wild-type prolactinomas (18). However, it is possible that increased presymptomatic detection of *MEN1* variants through improved genetic testing access in *MEN1* kindreds has increased the detection of phenocopies (ie, sporadic prolactinomas) that are comparatively mild and may have been undetected in the absence of *MEN1*-directed tumor surveillance. Based on the currently available evidence, management of MEN1-related PAs should follow sporadic PA guidelines (16).

### PRKAR1A

The *PRKAR1A* gene (Chr 17q24.2, encoding type 1 alpha regulatory subunit [RIα] of cAMP-dependent protein kinase–PKA) is a tumor suppressor gene that restrains basal cAMP–PKA signaling through sequestration of the catalytic subunits of protein kinase A. Loss of RIα removes this inhibitory control, leading to constitutive PKA activation that enhances cAMP response element-binding protein (CREB)-dependent transcription, cell-cycle progression, and proliferative signaling in pituitary cells (19). *PRKAR1A* was implicated as the causative gene in the majority of Carney complex (CNC) cases in 2000, with inactivating *PRKAR1A* variants observed to lead to increased PKA activity and widespread tumorigenesis (20). Most CNC tumors exhibit *PRKAR1A* loss of heterozygosity (LOH), with haploinsufficiency hypothesized as the mechanism of tumorigenesis in the remainder (19). CNC is associated with skin manifestations (lentigines, myxoma, blue nevi) in approximately 60% to 80%, primary pigmented nodular adrenal disease in ∼57%, and overt pituitary disease in ∼20% of individuals (21, 22). Notably, *PRKAR1A* variants are seldom found in people with isolated PAs, although the CNC phenotype may be missed at initial PA presentation and dedicated evaluation should be considered in people with young-onset acromegaly or gigantism.

The typical *PRKAR1A*-associated pituitary lesion is mammosomatotroph hyperplasia, although discernible PAs may occur and are most commonly somatotroph or mixed somatotroph–lactotroph microadenomas, with a single case series reporting the presence of isolated lactrotroph adenomas as well (22, 23). This leads to a spectrum of growth hormone (GH) excess presentations, ranging from asymptomatic GH excess in ∼35% of individuals to overt acromegaly in 20% of individuals, with median ages of onset at 25 and 26 years, respectively (22). GH excess in the setting of CNC has been reported from 9 years of age, with 26% of patients presenting with GH excess by the age of 18 years (22). Cushing's disease is rarely seen in CNC, and hypercortisolism in CNC is typically ACTH-independent and attributable to primary pigmented nodular adrenocortical disease (21).

### AIP

The *AIP* gene (Chr 11q13.2, encoding aryl hydrocarbon receptor [AHR] interacting protein) was first established as a cause of familial PA predisposition in 2006 (24). *AIP* encodes a molecular chaperone protein with several binding partners. The protein contains 3 tetratricopeptide repeats, each comprising an antiparallel pair of α-helices, and a final C-terminal α-helix that is critical for protein–protein interactions. Loss of structural integrity in this region is thought to impair binding to the AHR and phosphodiesterase 4A5, thereby compromising the tumor suppressive functions of AIP (25). Germline *AIP* variants account for up to 20% of FIPA families (26), and *AIP* is the gene most frequently found to harbor variants in multigene panel tests of patients with established or suspected familial PA conditions (27). Over 100 distinct heterozygous *AIP* variants have been reported, with an autosomal dominant pattern of inheritance, incomplete penetrance and variable expressivity (28).

*AIP*-related FIPA is characterized by earlier clinical presentations when compared against non-*AIP*-related familial and sporadic early-onset PAs (defined by disease onset ≤18 years or macroadenoma onset ≤30 years), with symptoms occurring approximately 8 years earlier (mean age of 19 years) and diagnosis established approximately 6 years earlier (mean age of 24 years) (29). In large international cohorts, 65% of *AIP* variant carriers have disease onset by age 18 years and 87% by 30 years (29). Family history may be negative for PAs due to either *de novo AIP* variants or incomplete penetrance resulting in seemingly sporadic cases. *AIP*-related PAs are most commonly somatotrophinoma (51%), followed by co-secreting mammosomatotrophinomas, typically exhibiting reduced treatment efficacy with resistance to somatostatin analogs and lower rates of surgical remission (28). The frequent treatment resistance of *AIP*-related PA is partly explained by typical markers of somatostatin resistance such as younger age of onset and low tumor expression of somatostatin receptor subtype 2, as well as a higher propensity for sparsely granulated histopathology (30). It has also been postulated that treatment resistance may be contributed to by mutant *AIP* impairing downstream action of the tumor-suppressive transcription factor *ZAC1* (zinc finger regulator of apoptosis and cell cycle arrest), a key mediator of the anti-proliferative effect of somatostatin receptors. Loss of functional *AIP* may blunt *ZAC1*-dependent growth inhibition despite preserved somatostatin receptor expression (31). Pituitary apoplexy has been proposed as a specific feature of *AIP*-related adenomas with a greater relative risk compared to PAs overall, not explained by their larger size or increased rate of macroadenomas compared to *AIP* wild-type cohorts (26, 29). However, this has not translated to higher rates of *AIP* variant detection when using pituitary apoplexy as an indication for genetic testing (32).

### CDKN1B

The *CDKN1B* gene (Chr 12p13.1, encoding cyclin-dependent kinase inhibitor p27^Kip1^) is a tumor suppressor gene, operating via modulation of cell cycle transition from G1 to S phase and thereby controlling cell proliferation and differentiation. In 2006, a frameshift variant of *Cdkn1b* was discovered to underlie MENX rats, which exhibit an overlapping MEN1/MEN2 syndrome, and a nonsense *CDKN1B* variant was concurrently detected in a woman with PA and a personal and family history compatible with MEN1 syndrome but without a detectable *MEN1* variant (33). Germline LOF *CDKN1B* variants are now considered to cause a similar but generally milder, later-onset MEN1-like phenotype, which has been termed “MEN4 syndrome’ (34). The condition is exceedingly rare, with *CDKN1B* variants found in only 0.07% of individuals with an MEN1 phenotype (35).

People with MEN4 syndrome present with PAs in ∼40% of cases, with a mean age of PA onset of 44 years (36). More recent pooled data from 74 genetically confirmed MEN4 cases estimate the cumulative risk of PA at approximately 23% by a median age of 34 years (range 5-79 years), somewhat earlier than older estimates, though this likely reflects ascertainment bias of historical case series of symptomatic presentations (37). *CDKN1B* variants are associated with a variety of PAs (38), with a potential predilection for pediatric corticotrophinomas (39). Compared with MEN1, where prolactinomas account for a large proportion of all PAs, early MEN4 data suggest a relative paucity of prolactinomas (37). Emerging genotype–phenotype data suggest that variant location within *CDKN1B* may influence pituitary risk. Variants in codons 94 to 96 are associated with a significantly higher risk of developing both primary hyperparathyroidism and PA, and indel variants appear to confer greater overall tumor risk compared with missense variants (37). As in MEN1, the approach to PA management should follow sporadic PA guidelines (36).

### GPR101

First reported in 2014, *GPR101*-containing Xq26.3 microduplications are the genetic hallmark of X-LAG, a rare, fully penetrant disorder from early childhood characterized by GH and prolactin excess with rapid linear growth (8). Duplications at Xq26.3 in X-LAG cause *GPR101* misexpression by reorganizing the 3D chromatin landscape rather than altering gene sequence. In the normal locus, *GPR101* sits within a topologically associating domain (TAD) whose invariant boundary insulates it from centromeric regulatory elements. The Xq26.3 microduplications implicated in X-LAG include and shift this invariant boundary, creating a neo-TAD that places *GPR101* in the same regulatory compartment as pituitary-active gene transcription enhancers. This enhancer-adoption mechanism forces ectopic activation of *GPR101*, which is highly permissive to incoming regulatory input, resulting in sustained overexpression that amplifies GH-secretory signaling and drives PA development in X-LAG (40). The speculated *GPR101* enhancer at the core of X-LAG was discovered in 2025 to be the *VGLL1* gene region by comparing existing Xq26.3 microduplication kindreds with an Australian family with an Xq26.3 duplication involving *GPR101* and the TAD invariant border but without any history of gigantism (41). Interestingly, one member of the Australian family with the seemingly non-penetrant Xq26.3 duplication developed an isolated prolactinoma, raising the possibility of *GPR101* dosage effects beyond the classical X-LAG mechanism, though this remains unproven (41). The essential role of the *VGLL1* region as the driver of *GPR101* misexpression in X-LAG has subsequently been corroborated by a bioinformatic approach of TAD enhancer mapping across pathogenic versus nonpathogenic Xq26.3 microduplications (42).

The clinical presentation of patients with X-LAG is distinct from other major causes of gigantism. Median age of onset is 18 months, with median diagnosis at 4 years and the typical first sign being proportional overgrowth in height and weight (43). There is a female predominance, and it is almost always sporadic, with only 3 familial cases previously identified and never father-to-daughter transmission (43, 44). The vast majority of X-LAG pituitary lesions are macroadenomas (82.1%), with the next largest subset being pituitary hyperplasia with no discrete adenoma (15.4%) (43). Prolactin co-secretion is common, present in almost 80% of patients (43). Trans-sphenoidal resection of the causative pituitary lesion is first-line management; however, a key biological challenge in X-LAG is that constitutive *GPR101* activation drives extremely high GH secretion, such that even minimal postoperative tumor remnants can sustain marked IGF-1 excess (45). Primary somatostatin receptor ligand therapy has been ineffective in achieving biochemical or tumor mass control when attempted, possibly due to *GPR101* constitutive activity overwhelming the inhibitory effect on SSTR2. Pegvisomant may be effective through its peripheral action as a GH receptor antagonist, as either monotherapy or combination therapy with somatostatin receptor ligands (46).

### SDHx

The succinate dehydrogenase (SDH) genes (*SDHx: SDHA*, *SDHB*, *SDHC*, *SDHD*, and *SDHAF2*), encoding SDH subunits and assembly factor 2 protein, are tumor suppressor genes. Germline LOF *SDHx* variants impair SDH enzymatic activity, causing accumulation of succinate and reactive oxygen species. This triggers a pseudohypoxic state via prolyl hydroxylase inhibition, which prevents hypoxia-inducible factor α (HIF-α) hydroxylation and degradation, ultimately driving angiogenesis, aberrant cellular proliferation, and tumorigenesis (47). *SDHx* variants are most frequently implicated in pheochromocytoma/paraganglioma (PPGL) development, but have also been described in PAs. *SDHx* variants follow autosomal dominant inheritance; however, *SDHD* and *SDHAF2* exhibit a parent-of-origin effect, with disease expression occurring almost exclusively after paternal inheritance (48, 49). The “3P (pheochromocytoma, paraganglioma, and PA) association syndrome’ (3PAs) was first coined in 2015 (50), although rare associations between PA and PPGL were previously described.

Variants in *SDHx* have been implicated in roughly one-third of reported cases of 3PAs in the literature, with a predisposition for larger and more invasive PAs (51). The average age at diagnosis of *SDHx*-related PA is 44 years (range 31 to 60) (51). *SDHx* variants are also associated with predisposition to renal cell carcinoma (RCC), gastrointestinal stromal tumor (GIST), and rarely all 4 *SDHx*-related tumor types (PPGL RCC, GIST, PA) may occur within the same kindred (52). Although *SDHx*-associated PAs are considered exceedingly rare, detected in only 1/309 (0.3%) PA operative specimens in a cohort study (53), the risk may be underestimated due to a lack of pituitary surveillance in *SDHx* protocols (54). Furthermore, *SDHx*-related PAs are most commonly prolactinomas, which tend to be medically treated without the need for surgery and hence with no opportunity for molecular testing of operative specimens. *SDHx*-related prolactinomas generally respond well to dopamine agonist therapy, although rare cases of treatment resistance and even pituitary carcinoma have been reported (55).

### MAX

The *MAX* gene (Chr 14q23.3) encodes a bHLH-LZ transcription factor that forms obligate heterodimers with both the MYC oncoprotein, promoting cell proliferation, and tumor-suppressive MXD family proteins, which restrain MYC-driven transcriptional programs. Loss of *MAX* disrupts these repressive MAX-MXD complexes, facilitating tumor formation via a 2-hit mechanism in which a heterozygous germline LOF variant is followed by somatic inactivation of the remaining allele, evidenced by LOH and absent MAX immunostaining in tumor tissue (56). As seen in *SDHD* and *SDHAF2*, there appears to be a parent-of-origin effect with disease expression typically occurring after paternal transmission (36, 51). Suspicious germline *MAX* variants have been variably associated with PA and other NETs, including PPGL, ganglioneuroma, and neuroblastoma, leading to the proposed terminology of “MEN5 syndrome’ (56). The primary manifestation of MEN5 syndrome is pheochromocytoma, which is often bilateral (57). *MAX* has become the most recently established PA predisposition gene through a 2025 mouse model corroborating prior clinical associations via the demonstration of PA development in *Max*-knockout mice (58).

A recent systematic review of MEN5 syndrome found that most patients had pheochromocytoma (88%), with PA the second most common NET (9%), followed by paraganglioma (8%), with a median age of PA diagnosis of 33.0 years (57). The typical *MAX*-related PA presentation is a prolactinoma or somatotrophinoma in individuals with a personal or family history of PPGL, responsive to dopamine agonist therapy (51).

## Emerging germline variants

In addition to genes with an established germline role in pituitary tumorigenesis, there are a number of emerging PA germline predisposition genes, including *CABLES1*, *PAM*, *CHEK2*, MMR genes, *PRLR*, *FGFR1*, *AHR*, *USP8*, *PRKACB* and *KDM1A* (Fig. 1). While there are data showing an association between these genes and PA development, there is a lack of multidimensional evidence demonstrating a causative, repeatable role for the genes in pituitary tumorigenesis, and thus these genes remain limited to research-based genetic testing only.

### CABLES1

*CABLES1* (Chr 18q11.2, encoding Cdk5 and Abl enzyme substrate 1) is a glucocorticoid-responsive regulator of cell cycle progression in corticotrophs. Under normal conditions, *CABLES1* expression induces a G1/S block, thereby suppressing proliferation. Loss of *CABLES1* expression has been observed in over half of corticotrophinomas with strong correlation with reduced p27^Kip1^, suggesting cooperative disruption of cell cycle inhibition (59). In 2017, Hernández Ramírez et al identified potentially pathogenic germline variants in *CABLES1* among 4 individuals (2 children and 2 adults) with Cushing's disease and pituitary macroadenomas (60). Taken together, these findings suggest that *CABLES1* may act as a tumor suppressor gene in corticotrophs, with its downregulation promoting PA development through failure of normal cell-cycle checkpoints, although this requires validation in independent Cushing's disease cohorts (4).

### CDH23

Biallelic pathogenic variants in *CDH23* (Chr 10q21.1, encoding a cell adhesion protein) have long been associated with Usher syndrome and non-syndromic deafness (61), whereas heterozygous germline variants were first detected in the PA setting in 2017 (62). CDH23 is thought to contribute to pituitary tumorigenesis by disrupting calcium-dependent cell–cell adhesion through extracellular cadherin domains (62). However, only isolated cases of sporadic PAs with germline *CDH23* variants have been reported since the seminal study (63, 64). There are no functional or larger-scale studies to date to confirm the putative role of *CDH23* variants in pituitary tumorigenesis.

### PAM

The *PAM* gene (Chr 5q21.1) encodes peptidylglycine α-amidating monooxygenase, which catalyzes C-terminal amidation, a modification that enhances the potency of many peptide hormones, including POMC-derived ACTH (65). *PAM* variants were first implicated in pituitary tumorigenesis and hormone hypersecretion in 2023, with deleterious *PAM* variants identified in kindreds with FIPA and patients with sporadic PAs (prolactinoma, corticotrophinoma, somatotrophinoma) recruited through multiple centers in the United States and Europe (66). This was corroborated by a 2023 Australian multicenter cohort study, confirming a high prevalence of suspicious *PAM* variants amongst functioning PAs (7/29 cases), and newly extending the phenotype to cyclical Cushing's disease and thyrotrophinomas (67). Given the relatively high prevalence of *PAM* germline variants but typically negative family history, we speculate that *PAM* variants might act as PA risk alleles with incomplete penetrance, though further studies are needed to clarify variant burden, penetrance and mechanistic pathways.

### CHEK2

*CHEK2* (Chr 22q12.1) encodes Checkpoint Kinase 2 (CHK2), a cell-cycle checkpoint regulator. It has an established role as a moderate-risk breast cancer predisposition gene and is included in germline breast cancer gene panel testing (68). Enrichment of germline *CHEK2* variants in the PA setting was first demonstrated in 2024, with suspicious *CHEK2* variants identified in 5/165 (3%) individuals from a mixed PA cohort. Pathogenic or likely pathogenic *CHEK2* variants were present at a higher frequency in people with PAs, compared with healthy controls (1.8% vs 0.5%; *P* = .049), suggesting that *CHEK2* may act as a risk allele for PA development, similarly to breast cancer (69). Supporting a potential role for *CHEK2* in PA formation, there are multiple case reports of carriers of *CHEK2* germline variants presenting with corticotrophinomas, somatotrophinomas, prolactinomas, nonfunctioning PAs, and even progression to pituitary carcinoma (70, 71). The clinical phenotype appears heterogeneous, with variable co-occurrence of non-pituitary tumors (71).

## MMR genes

MMR genes (*MLH1*, *MSH2*, *MSH6*, *PMS2* and *EPCAM*) may also contribute to PA development. In Lynch syndrome, caused by MMR gene variants, PAs appear overrepresented and often display aggressive behavior including pituitary carcinoma (72). PA samples from individuals with Lynch syndrome have demonstrated microsatellite instability and loss of the relevant MMR protein in immunohistochemistry (IHC), supporting a causal role for germline MMR gene variants in pituitary tumorigenesis (72). A recent next-generation sequencing (NGS) study observed a high burden of pathogenic germline variants across cancer predisposition genes, particularly in MMR genes, in individuals with suspected familial or sporadic PAs, with a relative rate of 1.44 compared to the general population; however, this did not reach statistical significance (73). Another recent study of mixed PAs demonstrated an association between reduced MMR expression and functioning corticotrophinomas and invasive prolactinomas (74).

### PRLR

*PRLR* (Chr 5p13.2) encodes the prolactin receptor, a multidomain class I cytokine receptor gene which activates JAK2–STAT5, PI3 K/Akt, and MAPK pathways to regulate transcription, proliferation and cell survival. Mouse models with *Prlr* deletion develop hyperprolactinemia, pituitary hyperplasia, and prolactinomas, indicating that chronic loss of PRLR signaling disrupts dopaminergic feedback and promotes lactotroph expansion (75, 76). Rare germline LOF *PRLR* variants have been detected in some but not all prolactinoma cohorts, possibly reflective of differing PA characteristics, with one cohort reporting an association with larger tumor size, cabergoline resistance, and higher levels of serum prolactin in prolactinomas with *PRLR* variants (77, 78).

### FGFR1

Pituitary embryogenesis genes have recently been evaluated to assess their potential contribution to PA development. *FGFR1* (Chr 8p11.23) encodes fibroblast growth factor receptor 1 (FGFR1). Expressed on pituitary precursor cells, FGFR1 is a tyrosine kinase receptor involved in pituitary embryogenesis through binding with FGF8 and activation of downstream pathways such as MAPK/ERK to promote physiological cell proliferation and anterior lobe expansion (79). *FGFR1* variants are implicated in 7-10% of cases of Kallmann syndrome and isolated hypogonadotropic hypogonadism, and have also been reported in combined pituitary hormone deficiency (80, 81). The *FGFR1*_D129A_ variant, previously reported in Kallmann syndrome (81), was recently identified in a man with a childhood-onset giant prolactinoma as well as his daughter with combined pituitary hormone deficiency, and was then found to be overrepresented in a mixed PA cohort compared to healthy controls (82). It is unclear how a single gene variant may produce discordant phenotypes of pituitary dysgenesis and neoplasia, but this may relate to inadequately differentiated cell lines that then proceed to unchecked cellular proliferation. Experimental data are required to explore this hypothesis.

### AHR

*AHR* (Chr 7p21.2) encodes aryl hydrocarbon, a ligand-activated transcription factor that normally sits in the cytoplasm bound to a chaperone complex that includes AIP (83). *AHR* is expressed across all adenohypophyseal cell types and restrains cellular proliferation through interaction with retinoblastoma protein and cyclin-dependent kinases. Experimental silencing of *AHR* increases cell proliferation in rat mammosomatotroph tumor models (83), and a human study identified a germline *AHR* variant in an individual with cyclical Cushing's disease (84).

### USP8

*USP8* (Chr 15q21.2) encodes ubiquitin-specific protease 8, which regulates EGFR recycling and is a well-established somatic driver gene in sporadic corticotrophinomas (85). In the sporadic setting, somatic *USP8* variants disrupt the USP8 14-3-3 binding motif, increasing USP8 catalytic activity and thereby enhancing EGFR deubiquitination (86). This, in turn, limits lysosomal degradation of EGFR and promotes its repeated recycling back to the plasma membrane, prolonging EGFR availability for signaling (85). Sustained EGFR activation drives MAPK signaling, which increases POMC transcription through a promoter region containing Tpit and Pitx1 binding sites, leading to elevated POMC mRNA, enhanced ACTH secretion, and corticotroph proliferation (87). In the germline setting, there have been 2 independent reports of germline *USP8* variants in 2 children with severe multisystem syndromic features (88), including early-onset Cushing's disease in 1 case (89).

### PRKACB

*PRKACB* (Chr 1p31.1, encoding catalytic subunit beta of PKA) participates in type I PKA holoenzymes and is activated when cAMP binds the regulatory subunits, releasing the catalytic subunit to phosphorylate downstream targets. This cAMP–PKA signaling axis regulates hormone synthesis, transcriptional programs, and cellular growth (90). A germline 1p31.1 triplication has been implicated in a single case of *PRKAR1A*-negative CNC with acromegaly (91).

### KDM1A

*KDM1A* (Chr 1p36.12, encoding lysine demethylase 1A) was first implicated in glucose-dependent insulinotropic polypeptide (GIP)-dependent primary bilateral macronodular adrenal hyperplasia, with germline inactivation and somatic LOH appearing to drive ectopic GIP receptor (GIPR) expression (92). Subsequent studies in PAs have not identified *KDM1A* sequence variants, but recurrent gross 1p deletions leading to *KDM1A* haploinsufficiency have been observed in somatotrophinomas (93). These deletions are associated with reduced *KDM1A* expression, increased GIPR expression, and a higher prevalence of paradoxical GH response following oral glucose loading, suggesting that epigenetic dysregulation at the GIPR locus may contribute to an alternative pathogenic route in a subset of somatotrophinomas (93).

## Oligogenic tumorigenesis

PA germline predisposition genes typically operate via the Knudson 2-hit hypothesis, whereby pituitary tumorigenesis occurs after the combination of an inherited or *de novo* germline variant with an acquired somatic second-hit in the other copy of the gene in question (94). Yet, there is a growing number of reported individuals with PAs who harbor suspected pathogenic germline and/or somatic variants in multiple genes, raising the possibility that digenic or oligogenic interactions may contribute to pituitary tumorigenesis (Table 1), though the pathogenicity of such dual variant findings have not been consistently demonstrated. The co-occurrence of variants in both candidate genes and established genes may cause doubt as to whether the variant in the candidate gene is truly pathogenic. This is enshrined in the American College of Medical Genetics and Genomics 2015 BP5 variant classification criterion, whereby finding a causative variant in a separate gene is considered evidence against a variant of uncertain significance being pathogenic (99). Nevertheless, reports of PAs associated with unequivocal pathogenic variants in more than one established gene support the hypothesis that multigenic mechanisms might contribute to pituitary tumorigenesis. The concept of oligogenic tumorigenesis is supported by clinical and basic evidence in other oncological settings (100, 101). Mechanistic studies involving cell line and animal models are required to further explore the hypothesis of oligogenic tumorigenesis in the pituitary.

**Table 1:** Examples of pituitary adenomas with concurrent germline and/or somatic variants suggestive of oligogenic tumorigenesis

Reference	Condition	Germline variant	Somatic variant
Pérez-Rivas, 2018 (95)	Nelson's syndrome	*AIP:* c.911G>A, p.(Arg304Gln)	*USP8:* c.2159C>A, p.(Pro720Arg)
Bao, 2022 (96)	Cushing's disease	*GPR101:* c.505G>C p.(Gly169Arg)	*USP8:* c.2155_2157delTCC, p.(S719del)
Sumislawski, 2022 (97)	Metastatic Cushing's disease	N/A	*TP53*: c.743G>A, p.(R248Q) *ATRX*: c.2044A>G, p.(N682D) *PTEN*: c.388C>T p.(R130Ter) *PTEN*: c.210-1G>A
De Sousa, 2024 (69)	Somatotrophinoma	*CHEK2*: c.1427C>T, p.(Thr476Met) *AIP*: c.490C>T, p.(Gln164Ter)	*MEN1*: c.496C>T, p.(Gln166Ter) *MEN1*: c.402dup, p.(Lys135GlnfsTer45)
Zainab, 2025 (98)	Cushing's disease	N/A	*MEN1:* c.1357C>T, p.(Gln453Ter) *PRKAR1A:* c.55G>T, p.(Glu19Ter) *TP53:* c.536A>G, p.(His179Arg)
Chasseloup, 2020 (39)	Cushing's disease	*CDKN1B*: c.356T>C, p.(Ile119Thr)	*USP8*: c.2159C>A, p.(Pro720Arg)

## A contemporary clinical approach to germline genetic testing

Germline genetic testing is now available across many pituitary centers to identify suspected familial PA conditions. It is ideally carried out in clinical settings with the combined endocrine, clinical genetics and genetic pathology expertise required to ensure appropriate patient selection, effective test methodologies and comprehensive pre- and post-test counselling.

Germline genetic testing should be considered where there is a high probability of detecting a germline gene variant (ie, young-onset PA—especially if <18 years, familial PA or where there is a personal and/or family history or non-pituitary tumors associated with an established PA predisposition gene), and the patient and/or their family are likely to benefit from detecting a germline variant (eg, to inform PA prognostication and management, extra-pituitary tumor surveillance, cascade testing and/or reproductive planning).

Given that there are multiple established PA predisposition genes with overlapping phenotypes, the test methodology increasingly utilized in the PA setting is NGS gene panel testing, which should ideally include all established predisposition genes (ie, *MEN1*, *PRKAR1A*, *AIP*, *CDKN1B*, *GPR101*, *SDHx*, and *MAX)*. Emerging predisposition genes should be specifically avoided in genetic testing in the clinical setting, given the uncertainty that a variant in such genes may introduce. Copy number variant (CNV) detection via either dedicated CNV-calling NGS pipelines or multiplex ligation-dependent probe amplification (MLPA) is critical to identify the pathogenic exon, whole gene, and larger deletions sometimes implicated in familial PA conditions (102). Microarray testing for the *GPR101/VGLL1-*containing Xq26.3 duplications that cause X-LAG should be considered in the specific scenario of early childhood-onset somatotrophinoma or somatotroph hyperplasia.

Pre-test counselling should be undertaken by experienced clinicians with comprehensive discussion of the indications for germline genetic testing, the process and potential results, and the implications of the specific variant on their PA prognosis, management and reproductive planning as relevant. Tumor surveillance should be arranged if the variant is identified in a gene associated with extrapituitary tumors (Table 2). Cascade testing should be offered to family members via the proband, taking care not to breach patient confidentiality. Unaffected relatives who test positive for the familial variant should undergo pituitary surveillance as outlined in Table 2. Where the causative gene is also associated with extrapituitary tumors—as is the case for several genes listed in Table 2, such as *MEN1*—both probands and carrier relatives should additionally undergo gene-specific surveillance for extrapituitary tumors in line with relevant international guidelines.

**Table 2:** Pituitary and extra-pituitary surveillance recommendations for germline pituitary adenoma predisposition gene carriers

Gene	Relevant international guideline or citation	Pituitary surveillance recommendations	Other tumor surveillance recommended*^a^*
*MEN1*	Brandi ML, et al (2025) (16)	Annual history, exam and visual field assessment from 10yo Serum prolactin and IGF-1 every 1-3 years from 10 yo MRI every 3-5 years from 15 yo	Y
*PRKAR1A*	Wasserman JD, et al (2025) (36)	Annual history, exam from puberty onset Serum IGF-1 from puberty onset	Y
*AIP*	No consensus guideline Korbonits M et al (2025) (103)	Annual history, exam from 4-30 yo, 5-yearly from 30-50 yo Visual field assessment from 10 yo Annual serum prolactin and IGF-1 between age 4-30 yo MRI every 5 years from 10 yo, ceasing at 30 yo if nil PA found	N
*CDKN1B*	No consensus guideline Chevalier B, et al (2024) (35)	Suggested as per MEN1 syndrome with additional screening for hypercortisolism	Y—as per MEN1 syndrome
*GPR101*	No consensus guideline Rostoyman L, et al (2015) (46)	No formal surveillance protocol established Regular serum IGF-1 measurement recommended in carrier infants who might have not yet manifested the condition	N
*SDHx*	Lussey-Lepoutre C, et al (2025) (54)	No formal surveillance protocol established Consider prolactin and other baseline pituitary hormonal evaluation Baseline imaging including skull base recommended	Y
*MAX*	Casey R, et al (2024) (104)	Surveillance guidelines exist for *MAX* related pheochromocytoma and paraganglioma but have not yet been established for pituitary adenomas	Y

Abbreviations: *AIP*, aryl hydrocarbon receptor-interacting protein; *CDKN1B*, cyclin-dependent kinase inhibitor 1B; *GPR101*, G protein-coupled receptor 101; IGF-1, insulin-like growth factor 1; *MAX*, MYC-associated factor X; *MEN1*, multiple endocrine neoplasia type 1; MRI, magnetic resonance imaging; PA, pituitary adenoma; *PRKAR1A*, protein kinase cAMP-dependent regulatory subunit type 1 alpha; *SDHx*, succinate dehydrogenase subunit genes; yo, years old.

^*a*^See the provided international guideline or citation for details regarding extra-pituitary tumor surveillance.

## Conclusions

PAs comprise a genetically heterogeneous group and our understanding of contributing germline variants is rapidly evolving. Established germline predisposition genes carry important implications for presentation, management, and tumor surveillance in individuals and their families, whilst a growing number of emerging predisposition genes warrant further investigation before adoption in clinical testing. Large-scale international registries, collaborative sequencing efforts, and further functional studies will be essential to validate these candidate genes and better define variant prevalence and clinical relevance.

## Data Availability

Data sharing is not applicable to this article as no datasets were generated or analyzed during the current study.

## Abbreviations

ACMG: American College of Medical Genetics and Genomics
ACTH: adrenocorticotropic hormone
AHR: aryl hydrocarbon receptor
AIP: aryl hydrocarbon receptor-interacting protein
bHLH-LZ: basic helix-loop-helix leucine zipper
CABLES1: cyclin-dependent kinase 5 and ABL enzyme substrate 1
CDH23: cadherin-related 23
CDKN1B: cyclin-dependent kinase inhibitor 1B
CHK2: checkpoint kinase 2
CNC: Carney complex
CNV: copy number variant
EGFR: epidermal growth factor receptor
EPCAM: epithelial cell adhesion molecule
FGFR1: fibroblast growth factor receptor 1
FIPA: familial isolated pituitary adenoma
GH: growth hormone
GIP: glucose-dependent insulinotropic polypeptide
GIPR: glucose-dependent insulinotropic polypeptide receptor
GIST: gastrointestinal stromal tumour
GPR101: G protein-coupled receptor 101
HIF-α: hypoxia-inducible factor alpha
IGF-1: insulin-like growth factor 1
KDM1A: lysine demethylase 1A
LOF: loss-of-function
LOH: loss of heterozygosity
MAX: MYC-associated factor X
MEN1: multiple endocrine neoplasia type 1
MLH1: mutL homolog 1
MLPA: multiplex ligation-dependent probe amplification
MMR: mismatch repair
MSH2: mutS homolog 2
MSH6: mutS homolog 6
MXD: MYC/MAX/MAD dimerization protein family
NET: neuroendocrine tumour
NGS: next-generation sequencing
PA: pituitary adenoma
PAM: peptidylglycine alpha-amidating monooxygenase
PitNET: pituitary neuroendocrine tumour
PKA: protein kinase A
PMS2: PMS1 homolog 2
POMC: pro-opiomelanocortin
PPGL: phaeochromocytoma/paraganglioma
PRKACB: protein kinase cAMP-dependent catalytic subunit beta
PRKAR1A: protein kinase cAMP-dependent regulatory subunit type 1 alpha
PRLR: prolactin receptor
PTH: parathyroid hormone
RCC: renal cell carcinoma
SDH: succinate dehydrogenase
SDHAF2: succinate dehydrogenase assembly factor 2
SDHx: succinate dehydrogenase subunit genes
SF1: steroidogenic factor 1
TAD: topologically associating domain
TPIT: T-box transcription factor
USP8: ubiquitin-specific protease 8
VGLL1: vestigial-like family member 1
X-LAG: X-linked acrogigantism
yo: years old
ZAC1: zinc finger regulator of apoptosis and cell cycle arrest
